# Unproved Remedies and Dr. Berridge

**Published:** 1881-12

**Authors:** P. P. Wells

**Affiliations:** Brooklyn, N. Y.


### UNPROVED REMEDIES AND DR. BERRIDGE.

P. P. Wells, M. D., Brooklyn, N. Y.

After the old story of the two knights, and the two-sided shield, putting myself and my friend Dr. Swan in the place of the knights, and "nosodes" in the place of the shield, and laying us both out helplessly by reason of our combat over the shield, Dr. B. modestly assumes the office of the third knight, and "to prevent the final catastrophe," whatever that might be, he "makes a few remarks." This is good of him, and the more so, as there is no knowing else what might have happened, especially as I, one of his assumed combatants, am wholly unconscious of having been in a fight at all with his other knight, and to this hour am equally unconscious of having received hurt at his hand. It would be a matter of regret to me if I had good reason for believing I had in any way injured my neighbor and colleague, as Dr. B. represents me to have done. In any event, it is to be earnestly hoped, that Dr. S. is as free from pain after his conflict as I feel myself to be, and that he is as grateful to the third knight for his unneeded benevolence as I am. But it is with the "few remarks" we are now to be chiefly concerned.

He first deals with my expressed regret that a declaration of the necessity that the dose of the dynamized drug be also that of a drug which has been *proved* on the healthy organism, was omitted in the "declaration of principles" issued by the "International Hahnemannian Association." His reason for this omission is a curiosity in its way. It was "implied." We submit that when a learned body undertakes an enunciation of the fundamental principles on which it rests its existence, as little as possible should be left to "implication." It leaves too large a range for loose imagination. Every man may affirm, and this loose method leaves him at liberty to do so, that this, that, or the other is implied, each claiming a new and different principle from the other, and nothing could be held under this rule as fixed. Nothing has so confirmed my sense of the need of this declaration as this paper of Dr. B. It cannot be uttered too soon if its authority is indeed now left to mere implication.

"It was not inserted" he says, "because what is true in it is already implied, and what is not implied is not true." Who shall tell us what is *not* implied? How as to nosodes? Is a practice by them implied? If so, then the more is the pity that this had not been plainly stated in the outset. It would have saved some of us the mortification of finding our names subscribed to this heresy, even by "*implication.*" To prove the implication, the Dr. makes a clear statement of the absolute necessity of this declaration, when he says: "Now the expression 'similars' necessarily implies the existence of two distinct groups of phenomena which are similar to each other, etc., * * * we must give remedies capable of producing similar symptoms, it implies that these similar symptoms must be first ascertained," etc. Just so, else how are we to know they are similar? Now as the implication has not proved strong enough to hold some of our best friends from resorting to unproved remedies, notably of unproved nosodes, can there be a stronger reason given for the express declaration, the absence of which we regretted? Our difficulty with the nosodes was—*unproved.* This and nothing more. Not with them as nosodes, but as *unproved.*

The next "remark" of the Doctor is a little extraordinary: "doubtless were our materia medica complete, were every medicinal substance exhaustively proved on healthy persons, nothing more would be required," etc. "But as matters at present stand, * * * we gladly avail ourselves of any light which can be thrown upon the case, either by symptoms obtained on the sick, or by clinical experience." If this has any thing to do with practice with unproved remedies we do not see it. If it be supposed to have, then the logic of it is this: As the materia medica is imperfect, and we are only partially acquainted with the drugs there recorded as proven, these being but partially proved, therefore we are to be justified in leaving that of which we know something, and instead of this take up that of which we know nothing. The controversy, if there be one, is between the known, partially known, if you please, and the absolutely unknown. The occasion of this controversy is not, on the one side, a protest against the freedom of any man to prescribe nosodes, if that is the best he can do, but against such a practice being thrust into our school of practice, and so we, who believe we have a better method, be made as a school responsible for it. Until the nosode is proved, it cannot be shown that a practice with it is homœopathic.

It would seem from Dr. B.'s attempt to answer my reference to *Syphilinum*, that his views and mine as to what constitutes a *proving* of a drug are not the same. I prefer my own, which are given in sufficient fullness in the paper to which he has attempted a reply. After giving his own, rather mistily, he acknowledges he does not know of any such record of the action of the syphilitic poison on the organism, as I have required as an essential part of every true proving, he asks: "Why should Dr. Wells stigmatize it as an unproved substance?" We answer, for the plain reason that there is no evidence that it has been proved as our law and philosophy require drugs to be before they can be incorporated into our materia medica or be accepted as legitimate agents for homœopathic practice. He insists upon it, the poison "*has been proved*," and "on healthy persons in the highest potiencies." Where is the record? In the absence of this, it is nothing to Dr. B. or myself as practitioners of homœopathy, if it has been proved a thousand times. In the absence of the record, it is altogether unavailable to us both in any prescription which is to be dominated by our law. This is our difficulty with this and all like agents. Not that they are nosodes, but that they are not known as they should and must be, before we can know that as the second factor in our problem of prescribing, it is similar to the first as our law requires it to be to constitute it a curative. Being thus unknown, if used clinically, it must be without and altogether without the pale of our law, and stand wholly with those agents employed by a bad empiricism. Not because "it is not in the Encyclopedia," but because unknown.

On page 520 of the Doctor's paper he seems to have failed of apprehending my argument as to the proving of this nosode. It was not that the persons suffering from the effects of this poison "had peculiarities of their own," but that those so suffering were likely, more than others, to be unsound in health, beyond the fact of the poisoning, and so the product to be proved is of necessity deficient in one of our cardinal requirements of drugs, before they can be accepted as agents to be proved, viz: *purity.* Unmixed with other agents or forces capable of modifying the effects of the drug proved. We require this of all drugs. Why not of all nosodes—*Syphilinum* included. In the paper to which the Doctor's is a reply I have treated this sufficiently at large to make more on the subject now unnecessary. The Doctor entitles his paper "*The Scientific Use of the Nosodes.*" Notwithstanding this, I fail to find in it aught that shows their use to be in any way or manner "*scientific.*" He has given no new principle for our guidance in their use. He has utterly failed to show their relationship to any law, homœopathic or otherwise, or given aught that can be perceived as an excuse for his so employing this word. It is a much abused word in these times, and the more is the pity. It is enough to sanctify whatever of eccentricity or extravagance in practice, if the sinner only uses this word freely enough, and deludes himself by its sweet sound into a belief that this word somehow redeems his vagaries from their true character and gives to them something of the character and worth of truth.

